# 

**Published:** 1872-03

**Authors:** 


### Contents of March Number.

### ORIGINAL DEPARTMENT.

ART. 1. Medical Society of the County of Albany. Semi-monthly meeting, Feb. 21st, 1872. Reported by James S. Bailey, M. D. ... 281
ART. 2. Case of Extra Uterine Fœtation. By Harvey Jewett, M. D., Canandaigua, N. Y., a paper read before the State Medical Society at their meeting in February, 1872 ... 287
ART. 3 Ligature of External Iliac. By S. A. Freeman, A. A. Surgeon U. S. A. ... 291
ART. 4. Ex-opthalmic Goitre, or Grave's Disease. Reported by J. W. Southworth, Toledo, Ohio ... 293
ART. 5. On the Recent Advances in the Theory of Vision. By H. Helmholtz. Translated by F. W. Abbott, M. D. ... 295

### MISCELLANEOUS.

Cerebro-Spinal Meningitis ... 301
The Use of Carbolized Catgut Ligatures ... 303
Iodized Milk ... 303
Tobacco ... 304
Disinfectant Treatment of Small-Pox ... 304
New Method of Extraction of Cataract ... 305
Treatment of Small-Pox ... 305
A Plan for Facilitating the Reduction of Strangulated Hernia by Taxis ... 306
New Operation for the Cure of Varicose Veins ... 307
An Instrument to Facilitate Skin-Grafting ... 308
Some of the Ill Effects of Bromide of Potassium ... 308

### EDITORIAL DEPARTMENT.

Appearance in Buffalo of Cereb o-Spinal Meningitis. * ... 309
Resolutions on the Death of Prof. Charles A, Lee. M. D. ... 311
Promotion ... 311
An Annual Exhibition for the American Medical Association ... 312
Elegant Medical Preparations ... 313

Books Reviewed:

Fireside Science: A Series of popular and scientific Essays upon subjects connected with every-day Life. By James R. Nichols, A. M., M. D. ... 313
War Department: Surgeons General's Office Circular, No. 3 Report of Surgical Cases in the Army, from 1865 to 1871. By Geo A Otis, M. D., Assistant Surgeon U. S. Army ... 313
Proceedings of the Royal Society ... 314
Transactions of the Twenty-Sixth Annual Meeting of the Ohio State Medical Society ... 314
Headaches, their Causes and their Cure. By Henry G Wright, M. D. etc. 314
On the Treatment of Pulmonary Consumption. By James Henry Bennet, M. D. ... 315
Transactions of the American Medical Association ... 315
Neuralgia and the Diseases that resemble it. By Francis E. Anstie, M. D. 3 5
A Practical Treatise on Bright's Diseases of the Kidneys By T. Grainger Stewart, M. D. etc ... 316
Transactions of the Medical Society of the State of Pennsylvania ... 316
Modern Medical Therapeutics. By Geo H. Napheys, A. M M. D. ... 317
Medical Thermometry and Human Temperature. By C A. Wunderlich, M. D. Translated by Edward Seguin, M. D ... 317
A Hand-Book of Therapeutics. By Sidney Ringer, M. D , etc ... 317
Boylston Prize Essay, 1871. By B. Joy Jeffries, A. M., M. D ... 318
The Works of Sir James Y. Simpson, Bart., M. D., D. C. L. Volume II. Edited by Sir W. G. Simpson, Bart., B. A. ... 318
Diagrams. By J. M. Toner, M. D ... 318
Books and Pamphlets Received ... 319

---

Address to the Graduating Class of the Buffalo Medical College, Feb., 1872. By Prof. James P. White, M. D. ... 321

---

* Our Readers will require great ingenuity, to make out what we designed to say about Cerebro-Spinal Meningitis ; as it is struck off without correction of proof.—Ed

